# Music learning motivation and students’ musical creative performance: the sequential mediating roles of creative self-efficacy and learning engagement

**DOI:** 10.3389/fpsyg.2026.1949126

**Published:** 2026-09-04

**Authors:** Dong Ding

**Affiliations:** Music College, Qilu Normal University, Jinan, Shandong, China

**Keywords:** creative performance, creative self-efficacy, learning engagement, music learning motivation, musical creativity, sequential mediation

## Abstract

This study examined whether autonomous music learning motivation was prospectively associated with university students’ demonstrated musical creative performance through temporally ordered indirect associations involving creative self-efficacy and learning engagement. A four-wave prospective design was employed with undergraduate students enrolled in music-related courses at one university in China, with 368 complete and valid cases retained for the primary analyses. Music learning motivation was assessed at Time 1, creative self-efficacy at Time 2, learning engagement at Time 3, and musical creative performance at Time 4 through a standardized composition task independently evaluated by three trained experts. Baseline creative performance and relevant background characteristics were controlled in the structural model. The proposed sequential indirect pathway from motivation to creative performance through creative self-efficacy and learning engagement was statistically supported (β = 0.052, 95% bootstrap CI [0.031, 0.078]), whereas the remaining direct association between motivation and final performance was not statistically significant after the mediators were included. The findings were consistent with a prospective pathway in which higher autonomous music learning motivation was associated with stronger subsequent creative self-efficacy and learning engagement, which in turn were associated with better expert-rated musical creative performance. However, the prospective observational design does not establish causal mediation, and broader generalization requires replication across institutions and learner populations.

## 1 Introduction

Music education involves more than the acquisition of technical knowledge and performance skills. It provides an important context in which learners explore ideas, express emotions, experiment with musical structures, and produce personally meaningful creative outcomes. Musical creativity may be demonstrated through composition, improvisation, arrangement, interpretation, and the transformation of existing material. These activities require learners not only to generate novel ideas but also to organize them into coherent, appropriate, and expressive musical products. Creativity is commonly understood as involving ideas or products that are both novel and appropriate within a particular domain (Amabile, 1996; Hennessey and Amabile, 2010). In educational settings, creative expression can vary in level and domain specificity, from personally meaningful creativity to more accomplished disciplinary performance (Kaufman and Beghetto, 2009). Musical creative performance can therefore be understood as a domain-specific manifestation of creativity that combines originality with musical coherence, expressive value, appropriateness, and technical completion.

Developing creative performance is not a purely technical process. Students with comparable musical knowledge and access to similar instructional resources may differ substantially in their willingness to explore alternatives, persist after unsuccessful attempts, revise incomplete work, and invest cognitive effort in musical creation. Motivation concerns why learners initiate and continue musical activity; self-efficacy concerns whether they believe that they can respond successfully to creative challenges; and engagement reflects the extent to which motivational intentions are accompanied by sustained learning behavior. Examining how these processes are prospectively related is important for explaining why some students develop high-quality creative work whereas others remain hesitant or minimally involved.

Music learning motivation is a particularly important starting point. From the perspective of self-determination theory, the quality of motivation is as important as its intensity. Learners may participate because they find music intrinsically interesting, because they personally value musical development, or because they feel pressured by grades, teachers, parents, or professional expectations. Intrinsic motivation and well-internalized forms of extrinsic motivation are relatively autonomous, whereas controlled motivation is characterized by external demands or internal pressure (Ryan and Deci, 2000, 2020). Autonomous motivation is especially relevant to musical creation because creative work involves uncertainty, experimentation, delayed improvement, and repeated revision.

Students who engage in music learning because they enjoy exploration or value creative development may be more willing to persist through uncertainty and to regard unsuccessful ideas as part of the learning process. By contrast, learners dominated by external pressure may focus on avoiding mistakes, satisfying assessment requirements, or reproducing established models. Existing research has linked motivation with persistence, practice, engagement, performance, and learning outcomes in music education (Chen, 2024; Su et al., 2024). Recent research in Chinese music-education contexts has also extended this line of inquiry to technology-supported learning, reporting relationships among music students’ perceptions of AI-supported learning, motivation, engagement, creativity, and learning success (Chen, 2025). Recent work has also connected basic psychological needs, intrinsic motivation, creative self-efficacy, and creative musicianship (Liu, 2025). These findings support the relevance of motivation while leaving open the question of how motivation is prospectively associated with independently evaluated creative performance through subsequent psychological and behavioral processes.

Creative self-efficacy represents one plausible intermediate process within this proposed prospective pathway. It refers to an individual’s belief in the capacity to generate creative ideas and perform creative tasks successfully (Tierney and Farmer, 2002). It is domain- and task-related rather than equivalent to general confidence. A learner may feel confident about reproducing a score or performing a familiar work while remaining uncertain about composing, improvising, or developing an original musical idea. Social cognitive theory proposes that efficacy beliefs influence goal selection, effort, persistence, strategy use, and reactions to difficulty (Bandura, 1997). Students with strong creative self-efficacy may approach difficult creative problems, continue working after setbacks, and regard incomplete ideas as material for further development.

Creative self-efficacy alone may be insufficient. Believing that one can produce creative work does not guarantee that sufficient time, attention, emotion, and strategy will be invested. Learning engagement provides a plausible bridge between efficacy beliefs and performance. Engagement is commonly conceptualized as behavioral, emotional, and cognitive (Fredricks et al., 2004). In music creation, behavioral engagement includes practice, persistence, attendance, task completion, and revision; emotional engagement includes interest, enjoyment, enthusiasm, and personal connection; and cognitive engagement includes strategic experimentation, reflection, self-monitoring, and integration of musical knowledge.

Musical creativity is iterative. Ideas are generated, tested, modified, combined, or rejected rather than appearing as complete products in a single attempt. Research on teacher–student interaction during composition indicates that novelty and appropriateness emerge dynamically through creative activity (Kupers and van Dijk, 2020). A student may possess promising ideas but fail to convert them into a coherent composition without sustained engagement. Conversely, high engagement can create repeated opportunities to develop and refine musical material.

Several methodological limitations remain in the literature. First, motivation, self-efficacy, engagement, and outcomes are frequently measured at a single time point, making temporal ordering difficult to evaluate. Second, many studies rely on self-reported creativity or perceived competence rather than directly assessed musical products. Self-report is useful for examining identity and confidence but does not establish the originality or quality of a composition. Third, limited research has examined engagement as a prospective process linking creative self-efficacy with subsequent musical creation. Fourth, common method variance can inflate associations when the same participant reports every construct in the same questionnaire.

The present study addresses these limitations through a four-wave prospective design. Music learning motivation is measured at Time 1, creative self-efficacy at Time 2, learning engagement at Time 3, and musical creative performance at Time 4. Students complete standardized composition tasks at baseline and at the final wave. Their products are anonymized and independently evaluated by trained music experts using a rubric informed by the Consensual Assessment Technique (Amabile, 1982; Hickey, 2001). Baseline creative performance and relevant musical-background variables are controlled.

The study integrates self-determination theory, social cognitive theory, and the multidimensional framework of learning engagement. It proposes that autonomous motivation supplies direction and personal value, creative self-efficacy supplies a task-specific belief in capability, and engagement supplies the active behavioral, emotional, and cognitive investment through which these resources become observable musical products.

Music Learning Motivation → Creative Self-Efficacy → Learning Engagement → Musical Creative Performance.

The study asks whether music learning motivation is associated with expert-rated musical creative performance, whether creative self-efficacy and engagement operate as separate mediators, and whether they form a sequential indirect pathway. It contributes by separating the variables across time, distinguishing creative self-efficacy from demonstrated creativity, controlling baseline creative performance, and using multi-source evidence rather than relying exclusively on student self-report.

## 2 Theoretical background and hypothesis development

### 2.1 Integrated theoretical perspective

Self-determination theory distinguishes autonomous from controlled motivation. Autonomous motivation arises when learners participate because they find an activity interesting, personally meaningful, or consistent with their values. Controlled motivation arises from rewards, sanctions, guilt, or compliance. Although either form may initiate behavior, autonomous motivation is generally more supportive of persistence, psychological ownership, flexibility, and deep learning (Ryan and Deci, 2020). Musical creation is an especially relevant setting because students must tolerate ambiguity and make choices that cannot be reduced to one correct answer.

Social cognitive theory explains how efficacy beliefs influence action. Self-efficacy affects the goals individuals choose, the effort they invest, their persistence in difficulty, and their interpretation of failure (Bandura, 1997; Zimmerman, 2000). Creative self-efficacy is the task-specific belief that one can generate, develop, revise, and complete original musical ideas. It links a general motivational orientation to an expectation that successful creative action is possible.

The multidimensional framework of engagement describes the enactment of motivation and efficacy. Behavioral engagement concerns participation and persistence; emotional engagement concerns interest and affective involvement; cognitive engagement concerns strategic investment, reflection, and self-regulation (Fredricks et al., 2004). These dimensions represent the work through which a learner’s interest and confidence become a developed musical product.

### 2.2 Music learning motivation and musical creative performance

Musical creative performance requires learners to generate original material while maintaining coherence, appropriateness, and expression. Motivated learners are more likely to initiate exploratory processes, consider multiple possibilities, and remain involved long enough for initial ideas to be refined. Autonomous motivation may also encourage artistic risk-taking because unsuccessful attempts are interpreted as part of learning rather than as evidence of inability.

Although motivation is expected to show a positive total association with creative performance, its association may be partly indirect. A learner may value music but doubt the ability to compose, or may feel motivated but fail to invest sufficient time and strategy in a task. The direct association therefore represents the overall relationship before the proposed intermediate pathways are considered.

*H1:* Music learning motivation is positively associated with students’ musical creative performance.

### 2.3 Music learning motivation and creative self-efficacy

Motivated learners are more likely to attempt challenging activities, seek feedback, revise incomplete work, and accumulate mastery experiences. These experiences are among the strongest sources of self-efficacy. Autonomous motivation may also change the interpretation of failure: an ineffective musical idea can be understood as information for improvement rather than proof of a fixed lack of talent.

Creative self-efficacy is therefore expected to develop when students value music learning and remain involved long enough to recognize their capacity for improvement. This expectation is consistent with research linking music-related self-efficacy, motivation, and engagement (Chen, 2024).

*H2:* Music learning motivation is positively associated with students’ creative self-efficacy.

### 2.4 Creative self-efficacy and musical creative performance

Creative work contains uncertainty. Students may need to compare alternative harmonizations, restructure a passage, or discard an idea after substantial effort. Those with stronger creative self-efficacy are more likely to view these demands as manageable, generate multiple alternatives, take artistic risks, and persist after setbacks. Tierney and Farmer (2002, 2011) showed that creative self-efficacy is distinct from general efficacy and is associated with creative performance over time. Creative self-efficacy has also been associated with students’ engagement in creative activity and creativity-related outcomes in educational contexts (Beghetto, 2006), supporting the relevance of domain-specific efficacy beliefs for musical creation.

The present study measures efficacy through self-report but evaluates performance independently. This separation makes it possible to test whether confidence is associated with demonstrated rather than merely perceived creativity.

*H3:* Creative self-efficacy is positively associated with students’ musical creative performance.

### 2.5 Music learning motivation and learning engagement

Autonomous motivation encourages students to participate because they value the process, not merely because participation is required. In musical creation, this distinction matters because a minimally acceptable assignment may be completed quickly, whereas a developed work requires repeated listening, evaluation, experimentation, and revision.

Motivation may influence emotional involvement, behavioral persistence, and cognitive strategy use. Learners who find music meaningful are expected to devote effort beyond minimum requirements and to use feedback and reflection more actively.

*H4:* Music learning motivation is positively associated with students’ learning engagement.

### 2.6 Creative self-efficacy and learning engagement

Students are more likely to invest effort when they believe that their actions can lead to improvement. Strong creative self-efficacy can reduce fear of failure, support enjoyment of experimentation, and encourage the use of demanding strategies. Low-efficacy learners may disengage even when they initially value the task because difficulty is interpreted as evidence that success is unattainable.

Creative self-efficacy is therefore expected to provide one prospective link between autonomous motivation and subsequent active involvement. It provides the capability judgment that can make sustained engagement psychologically reasonable.

*H5:* Creative self-efficacy is positively associated with students’ learning engagement.

### 2.7 Learning engagement and musical creative performance

Creative products improve through iteration. Behavioral engagement creates opportunities to revise; emotional engagement supports personal investment and expression; cognitive engagement supports analysis of relationships among melody, rhythm, harmony, texture, and form. Engaged learners are more likely to identify weaknesses, test alternatives, and integrate knowledge into artistic decisions.

Accordingly, engagement should be reflected in expert judgments of thematic development, coherence, expression, technical completion, and overall creative quality.

*H6:* Learning engagement is positively associated with students’ musical creative performance.

### 2.8 Mediating roles of creative self-efficacy and learning engagement

Creative self-efficacy may mediate the relationship because motivation supplies reasons to participate, while efficacy supplies a task-specific expectation of successful action. A motivated learner who continues to doubt creative capability may avoid open-ended creation. When motivation contributes to stronger efficacy beliefs, the learner may be more willing to attempt and refine original ideas.

*H7:* Creative self-efficacy mediates the relationship between music learning motivation and musical creative performance.

Learning engagement may also mediate the relationship. Autonomous motivation can directly encourage behavioral, emotional, and cognitive investment, which provides the repeated activity needed to develop a musical product.

*H8:* Learning engagement mediates the relationship between music learning motivation and musical creative performance.

### 2.9 Sequential mediation

**Figure 1:**
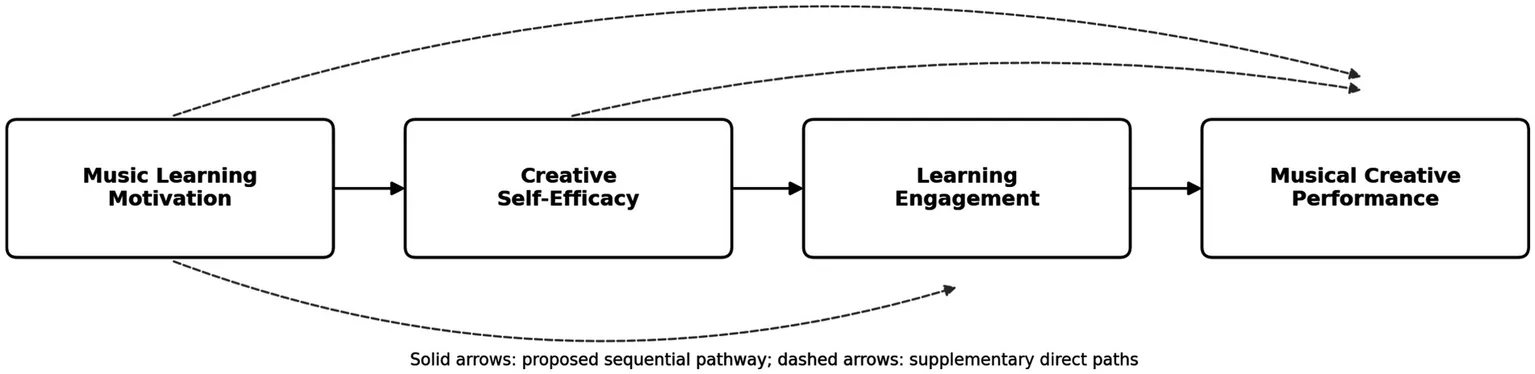
Proposed sequential mediation model. Control variables are omitted for visual clarity.

The integrated model proposes a temporally ordered sequence in which motivation is associated with subsequent creative self-efficacy, efficacy is associated with later learning engagement, and engagement is associated with final creative performance. The sequence distinguishes reasons for action, perceived capability, active investment, and demonstrated outcome. It does not assume that any one component is sufficient on its own or establish that the sequence is causal (see Figure 1).

*H9:* Creative self-efficacy and learning engagement sequentially mediate the relationship between music learning motivation and musical creative performance.

## 3 Methods

### 3.1 Research design

The study adopted a four-wave prospective design. The measurement waves were separated by approximately three weeks so that the predictor, mediators, and outcome were not collected simultaneously. At Time 1, participants completed measures of music learning motivation, demographic and musical-background variables, and a baseline musical creation task. At Time 2, creative self-efficacy was assessed. At Time 3, learning engagement was assessed. At Time 4, participants completed a parallel creation task, and their products were evaluated by independent experts.

The design did not manipulate motivation or randomly assign participants. It therefore tested temporally ordered associations and indirect pathways rather than definitive causal effects. Baseline musical creative performance was controlled to reduce the possibility that initial creative ability explained the observed relationships. The combination of student-reported psychological measures, standardized products, and blind expert ratings was intended to reduce common method bias.

MLM(T1) → CSE(T2) → LE(T3) → MCP(T4)

### 3.2 Participants and sampling

Participants were undergraduate students enrolled in music-related courses at Qilu Normal University in Jinan, Shandong Province, China. An initial recruitment target of approximately 420 students was established to retain at least 350 usable cases after anticipated four-wave attrition.

A total of 424 students participated at Time 1, and 368 complete and valid cases were retained for the primary analyses. Participants were eligible if they were enrolled in a participating course, provided informed consent, completed the Time 1 motivation assessment, completed the creation tasks required for the primary analysis, and had no uncorrected condition that prevented completion of the listening or notation tasks. Participants were excluded for invalid matching codes, failure of two attention checks, use of externally generated material, use of generative AI during a supervised task, excessive missingness, or submission of a composition that failed the minimum task requirements.

Background variables included age, gender, academic year, music-major status, years of formal music training, principal instrument, weekly practice time, previous composition or arrangement experience, digital music software experience, ensemble participation, improvisation experience, and previous use of generative AI. Baseline musical creative performance, age, gender, music-major status, years of formal music training, weekly practice time, and previous composition or arrangement experience were treated as the principal covariates. The covariate set was defined on substantive grounds because these variables represent pre-existing demographic or musical-experience characteristics that could plausibly contribute to individual differences in subsequent creative performance. In the fully adjusted structural model, all seven covariates were retained regardless of their individual statistical significance and were specified as predictors of Time 4 musical creative performance. Baseline creative performance was treated as the theoretically most important covariate because it directly adjusted for pre-existing differences in demonstrated musical creative ability.

### 3.3 Procedure

#### 3.3.1 Pilot phase

Questionnaire wording, task instructions, two parallel musical motifs, and the expert rubric were piloted with approximately 60 students who were not included in the main sample. Ten students completed cognitive interviews. Five music educators or composers evaluated the motifs for difficulty, melodic complexity, rhythmic density, tonal clarity, familiarity, and development potential. Motifs were retained only when they offered comparable opportunities for creative development and did not differ meaningfully in perceived difficulty.

#### 3.3.2 Time 1

Participants provided consent, generated a confidential identification code, completed the background questionnaire and the 12-item music learning motivation scale, and completed a 60-min baseline creation task. They were informed that the work would be evaluated for research but would not affect course grades. The mediation hypotheses were not disclosed.

#### 3.3.3 Time 2

Approximately three weeks later, participants completed the six-item creative self-efficacy measure. Instructions asked them to consider recent experiences in composition, improvisation, arrangement, or creative musical problem-solving. One instructed-response attention check was embedded but excluded from scoring.

#### 3.3.4 Time 3

Approximately three weeks after Time 2, students completed the 15-item music learning engagement measure, referring to their participation during the preceding four weeks. Where available, supplementary behavioral indicators were collected from course or platform records, including task submissions, punctuality, revisions, attendance, and access to materials.

#### 3.3.5 Time 4

Approximately three weeks after Time 3, participants completed the final 60-min composition task. Motif A and Motif B were counterbalanced using AB and BA sequences. All files were assigned random identifiers, and Time 1 and Time 4 products were mixed into a single rating set so that experts were blind to participant identity, time point, task sequence, and questionnaire responses.

### 3.4 Measures

#### 3.4.1 Music learning motivation

Autonomous music learning motivation was assessed using 12 context-specific items developed for the present study rather than adopted verbatim from an existing standardized instrument. Item generation was guided by self-determination theory, particularly the distinction between intrinsic motivation and identified regulation (Ryan and Deci, 2000, 2020). Six items represented enjoyment, curiosity, and intrinsic interest, whereas six represented personally endorsed value and importance. All items were contextualized to university music-learning activities and followed the stem, “I participate actively in music learning because…”. Responses ranged from 1 (strongly disagree) to 7 (strongly agree). A second-order autonomous-motivation factor was specified above the two first-order dimensions.

1. I enjoy discovering new possibilities in music.
2. I find music learning interesting in itself.
3. I feel satisfied when I understand a new musical idea.
4. I enjoy experimenting with different musical expressions.
5. Music learning activities are personally enjoyable.
6. I feel curious about how musical ideas can be developed.
7. Developing my musical ability is personally important to me.
8. Music learning contributes to goals that I value.
9. Learning music is meaningful for my personal development.
10. Improving my capacity to create music is worth the effort.
11. Music learning helps me express ideas that are important to me.
12. I invest effort because I recognize the value of musical development.

#### 3.4.2 Creative self-efficacy

Creative self-efficacy was assessed using six context-specific items developed for the present study rather than adopted verbatim from an existing standardized instrument. Item generation was informed by the domain- and task-specific conceptualization of creative self-efficacy described by Tierney and Farmer (2002, 2011) and Beghetto (2006), with the content specifically contextualized to musical creation. The items assessed original idea generation, development of musical material, creative problem solving, coherence, revision, and expressive realization rather than general academic or performance confidence. Responses ranged from 1 (strongly disagree) to 7 (strongly agree).

1. I am confident that I can generate original musical ideas.
2. I can develop a simple musical idea into a more complete work.
3. I can find creative solutions when I encounter difficulties in music creation.
4. I am capable of producing music that is both original and coherent.
5. I can revise an initially weak musical idea until it becomes effective.
6. I am confident that I can express my own ideas through musical creation.

#### 3.4.3 Music learning engagement

Music learning engagement was assessed using 15 context-specific items developed for the present study rather than adopted verbatim from an existing standardized instrument. Item generation was guided by the multidimensional engagement framework of Fredricks et al. (2004), with five items representing behavioral, emotional, and cognitive engagement, respectively. All items were contextualized to university music learning and creative activity and were rated on a 7-point scale ranging from 1 (strongly disagree) to 7 (strongly agree). A second-order engagement factor was specified above the three first-order dimensions.

##### 3.4.3.1 Behavioral engagement

- I participate actively in music-learning activities.
- I complete assigned tasks on time.
- I continue working when a music task becomes difficult.
- I devote regular effort to improving my musical work.
- I revise my work rather than stopping after the first attempt.

##### 3.4.3.2 Emotional engagement

- I feel interested during music-learning activities.
- I enjoy exploring different musical possibilities.
- I feel emotionally involved when working on music tasks.
- I look forward to activities involving musical creation.
- I feel satisfied when my musical work improves.

##### 3.4.3.3 Cognitive engagement

- I compare possible solutions before making a musical decision.
- I reflect on whether my choices achieve the intended effect.
- I use feedback to revise and improve my work.
- I connect new music concepts with previous knowledge.
- I monitor the coherence and expressive quality of my work.

#### 3.4.4 Musical creative performance

Musical creative performance was assessed through standardized creation tasks and blind expert ratings. Each product was rated from 1 to 7 on originality, musical coherence, thematic development, expressiveness, technical completion, and overall creative value. The first five dimensions captured analytically distinguishable characteristics of the musical product, whereas overall creative value represented a holistic expert judgment of the work as an integrated creative product. The primary score was the mean of the six dimensions across three raters. The Time 1 score represented baseline performance, and the Time 4 score represented the principal outcome. Because the holistic overall-creative-value rating partly incorporated judgments that overlap conceptually with several analytic dimensions, the dimensional structure of the six ratings was evaluated separately and a sensitivity analysis was conducted using an alternative five-dimension composite that excluded overall creative value.

*MCPᵢ = Σ Scoreᵢᵣd/18*, for three raters and six dimensions

### 3.5 Standardized creation tasks

Two original eight-measure motifs were developed in 4/4 meter at a moderate tempo. They had comparable pitch ranges, event density, tonal clarity, and development potential, avoided recognizable melodies, and were not tied to a commercial style. Participants were instructed to develop the motif into a complete 24–32-measure composition while retaining its recognizability.

Participants could use repetition, variation, sequence, rhythmic transformation, reharmonization, textural development, register change, instrumentation, fragmentation, and recombination. They were required to develop the material in at least two musical dimensions. All participants worked under supervision for 60 min using the same version of MuseScore or technically equivalent software, standardized devices, and a common audio-rendering library. Internet access, collaboration, import of prior MIDI files, and generative AI were prohibited.

The task instruction was: “You will be given an eight-measure musical motif. Develop this motif into a complete musical composition of 24–32 measures. The original motif should remain recognizable, but you may transform it through repetition, variation, sequence, rhythmic change, reharmonization, textural development, register change, or instrumentation. Your final work should demonstrate both originality and musical coherence. You have 60 minutes”.

### 3.6 Expert rating procedure

Three experts were recruited, each holding a postgraduate qualification or equivalent professional experience in composition, music education, theory, or digital music, with at least five years of teaching or professional experience and no direct instructional relationship with the participants. They completed calibration using 20 pilot compositions, but formal ratings were independent. The use of domain-expert judgment was consistent with the Consensual Assessment Technique and prior work on the assessment of creativity in musical compositions (Amabile, 1982; Hickey, 2001; Byrne et al., 2003). A two-way random-effects intraclass correlation coefficient for absolute agreement based on the mean of three raters, ICC(2,3), was calculated (see Table 1).

**Table 1:** Expert rating dimensions and operational definitions.

Dimension	Operational definition	Scale
Originality	Novel, distinctive, and non-mechanical treatment of musical material	1–7
Musical coherence	Unity, continuity, and intelligible organization of the work	1–7
Thematic development	Meaningful transformation, expansion, contrast, or integration of the motif	1–7
Expressiveness	Clarity and effectiveness of emotional or artistic intention	1–7
Technical completion	Internal consistency of rhythm, notation, harmony, texture, form, and ending	1–7
Overall creative value	Holistic judgment of originality, appropriateness, coherence, and artistic success	1–7

### 3.7 Translation and preliminary validation

Because the study-specific measures were developed for use in a Chinese university music-learning context, the item pools underwent a structured development and preliminary validation process. Initial items were generated with reference to the relevant theoretical frameworks and prior empirical literature and were reviewed by specialists in educational psychology and music education for conceptual relevance, clarity, and contextual appropriateness. Five experts subsequently evaluated item relevance and clarity, and items with item-level content-validity indices below 0.78 were revised or removed. To check conceptual equivalence between the Chinese items administered to participants and the English item formulations used for reporting, the wording was independently reviewed by two bilingual researchers, reconciled, and back-translated by a third researcher. Cognitive interviews were then used to identify ambiguous or unnatural wording, followed by pilot testing in a separate sample that was not included in the principal study.

### 3.8 Data analysis

Analyses were conducted in R and Mplus. Data were screened for duplicate cases, invalid codes, missing values, attention-check failure, unusual completion times, non-normality, and invalid task files. The primary structural analyses used the 368 complete and valid cases with data available for all focal variables. To assess sensitivity to missing-data handling, the structural model was additionally estimated using full-information maximum likelihood with all available longitudinal observations.

Cronbach’s alpha, McDonald’s omega, composite reliability, and average variance extracted were calculated for the self-report constructs. Confirmatory factor analysis compared the hypothesized higher-order measurement model with models that combined adjacent constructs and with a single-factor model. The higher-order model specified autonomous music learning motivation above intrinsic motivation and identified regulation and specified learning engagement above behavioral, emotional, and cognitive engagement. Because the higher-order autonomous-motivation factor was defined by only two first-order factors, the two unstandardized second-order factor loadings were constrained to equality for model identification. Their standardized loadings were permitted to differ because standardization also reflects differences in the variances of the first-order factors. The six expert-rated musical creative performance dimensions were additionally evaluated using a one-factor CFA to examine whether they could be represented by a common performance construct. Fit was evaluated using χ^2^, CFI, TLI, RMSEA, and SRMR. CFI and TLI values close to or above 0.90 and RMSEA and SRMR values close to or below 0.08 were treated as general guidelines rather than absolute rules.

The structural model included paths from music learning motivation to creative self-efficacy, engagement, and final creative performance; from creative self-efficacy to engagement and performance; and from engagement to performance. Baseline musical creative performance, age, gender, music-major status, years of formal music training, weekly practice time, and previous composition experience were retained as covariates predicting Time 4 musical creative performance. Gender, music-major status, and previous composition experience were entered as binary indicator variables; the original analysis-dataset coding was retained. Indirect effects were evaluated with 5,000 bias-corrected bootstrap resamples. An indirect effect was supported when its 95% interval excluded zero.

Participants were nested within 12 classes within a single institution. Class-level clustering was examined using intraclass correlations and design effects, and the principal structural model was additionally estimated using class-clustered robust standard errors as a sensitivity analysis. Further sensitivity analyses examined full-information maximum likelihood estimates, exclusion of highly experienced composers, task sequence, a parsimonious covariate specification controlling only baseline creative performance, and an alternative five-dimension musical creative performance score excluding overall creative value. The exploratory music-major versus non-music-major multigroup comparison reported in the submitted version was not retained because structural group comparisons require prior establishment of measurement invariance and were not central to the study hypotheses.

### 3.9 Ethics and open science

The study involving human participants was reviewed and approved by the Ethics Committee of Qilu Normal University. The committee did not issue a separate approval number for this study. All procedures were conducted in accordance with applicable institutional guidelines and ethical standards. Participants received written information regarding the purpose and procedures of the study, confidentiality, voluntary participation, and their right to withdraw without academic penalty. Written informed consent was obtained from all participants before data collection. Participation in the study and the expert ratings of students’ musical products did not affect their course grades.

## 4 Results

### 4.1 Participant flow and preliminary screening

A total of 424 students completed Time 1. At Time 2, 407 completed creative self-efficacy; at Time 3, 389 completed learning engagement; and at Time 4, 374 completed the final task. Six cases were excluded after screening: two failed attention checks, two submitted products below the minimum requirements, and two had invalid matching codes. The complete-case sample was 368, corresponding to 86.8% retention.

The sample included 250 women and 118 men, with a mean age of 20.12 years (SD = 1.02). Two hundred and twenty-two participants (60.3%) were in music-related majors. Mean formal music training was 4.53 years (SD = 2.98), weekly practice was 4.36 h (SD = 2.81), and 179 students (48.6%) reported previous composition or arrangement experience. Completers and non-completers did not differ significantly in motivation, baseline creative performance, training, or gender. A logistic regression of completion on baseline variables was not significant, χ^2^(7) = 8.41, *p* = 0.298 (see Table 2).

**Table 2:** Participant flow across four waves.

Stage	Measurement	*N*	Retention
Time 1	Motivation, background, baseline task	424	100.0%
Time 2	Creative self-efficacy	407	96.0%
Time 3	Learning engagement	389	91.7%
Time 4	Final creation task	374	88.2%
Final sample	Complete and valid cases	368	86.8%

Item-level missingness ranged from 0.2 to 1.8%. Absolute skewness ranged from 0.08 to 0.54 and absolute kurtosis from 0.11 to 0.82. Variance inflation factors ranged from 1.08 to 1.74. Five multivariate outliers were retained because no invalid response process or administrative error was identified; excluding them did not change the conclusions.

### 4.2 Equivalence of tasks and inter-rater reliability

At Time 1, Motif A (M = 4.02, SD = 0.84) and Motif B (M = 3.97, SD = 0.86) did not differ, t(366) = 0.54, *p* = 0.589, d = 0.06. At Time 4, AB and BA sequences also did not differ, t(366) = −0.43, *p* = 0.668. Task sequence did not interact with baseline performance, F(1, 364) = 0.42, *p* = 0.518.

The overall inter-rater reliability was good, ICC(2,3) = 0.89, 95% CI [0.86, 0.91]. Dimension-level ICCs ranged from 0.82 to 0.90, indicating good agreement among the three expert raters. Because ICC assesses rater agreement rather than the dimensional structure of the performance outcome, the common-factor structure of the six rating dimensions was examined separately by CFA (see Table 3).

**Table 3:** Inter-rater reliability of expert ratings.

Dimension	ICC(2,3)	95% CI	Interpretation
Originality	0.83	[0.79, 0.86]	Good
Musical coherence	0.87	[0.84, 0.89]	Good
Thematic development	0.85	[0.82, 0.88]	Good
Expressiveness	0.82	[0.78, 0.85]	Good
Technical completion	0.90	[0.88, 0.92]	Excellent
Overall creative value	0.88	[0.85, 0.90]	Good
Composite	0.89	[0.86, 0.91]	Good

### 4.3 Baseline and final performance

Mean musical creative performance increased from 4.00 (SD = 0.85) at Time 1 to 4.20 (SD = 0.90) at Time 4, t(367) = 4.98, *p* < 0.001, dz. = 0.26. Because the study did not include a no-instruction control condition, the average increase was not interpreted as a causal course effect. Baseline performance was controlled in all principal outcome models.

### 4.4 Reliability and measurement model

Music learning motivation showed high internal consistency (α = 0.927, ω = 0.930). Creative self-efficacy showed α = 0.909 and ω = 0.911. Engagement showed α = 0.936 and ω = 0.938. Composite reliability values ranged from 0.910 to 0.937 and average variance extracted from 0.58 to 0.64. Standardized item loadings ranged from 0.69 to 0.86 and were significant at *p* < 0.001. Heterotrait-monotrait ratios ranged from 0.42 to 0.72 (see Table 4).

**Table 4:** Reliability and convergent validity.

Construct	Items	α	ω	CR	AVE
Music learning motivation	12	0.927	0.930	0.928	0.61
Creative self-efficacy	6	0.909	0.911	0.910	0.64
Learning engagement	15	0.936	0.938	0.937	0.58

The hypothesized higher-order measurement model showed good fit: χ^2^(486) = 641.83, *p* < 0.001, CFI = 0.963, TLI = 0.959, RMSEA = 0.030, 90% CI [0.023, 0.036], SRMR = 0.041. Under the equality constraint imposed on the unstandardized second-order loadings for identification, the corresponding standardized loadings were 0.92 and 0.89 for intrinsic motivation and identified regulation on autonomous motivation, respectively. Standardized second-order loadings were 0.87, 0.90, and 0.93 for behavioral, emotional, and cognitive engagement on the higher-order engagement factor. A model combining creative self-efficacy and engagement fit substantially worse (CFI = 0.837, RMSEA = 0.063), and a single-factor model showed poor fit (CFI = 0.581, RMSEA = 0.116). A separate one-factor CFA of the six Time 4 expert-rated performance dimensions also showed good fit, χ^2^(9) = 17.62, *p* = 0.040, CFI = 0.991, TLI = 0.984, RMSEA = 0.051, 90% CI [0.010, 0.086], SRMR = 0.018. Standardized loadings ranged from 0.74 to 0.90, providing additional support for representing the six ratings by a common musical creative performance construct. Detailed factor loadings are reported in Supplementary Table S4 (see Table 5).

**Table 5:** Measurement model comparison.

Model	χ^2^	df	CFI	TLI	RMSEA	SRMR
Hypothesized higher-order model	641.83	486	0.963	0.959	0.030	0.041
CSE and engagement combined	1198.46	489	0.837	0.824	0.063	0.078
Single-factor model	2941.72	495	0.581	0.553	0.116	0.129

### 4.5 Descriptive statistics and correlations

Music learning motivation correlated positively with creative self-efficacy (r = 0.453), engagement (r = 0.398), and final creative performance (r = 0.308), all *p* < 0.001. Creative self-efficacy correlated with engagement (r = 0.545) and final performance (r = 0.359), while engagement correlated with final performance (r = 0.381). Baseline performance was strongly related to final performance (r = 0.607) but only weakly related to the self-report constructs (see Table 6).

**Table 6:** Means, standard deviations, and correlations.

Variable	M	SD	1	2	3	4	5
1. Motivation	5.14	0.66	—				
2. Creative self-efficacy	4.89	0.73	0.453***	—			
3. Engagement	4.86	0.66	0.398***	0.545***	—		
4. Baseline performance	4.00	0.85	0.090	0.058	0.021	—	
5. Final performance	4.20	0.90	0.308***	0.359***	0.381***	0.607***	—

*N* = 368. *** *p* < 0.001.

### 4.6 Structural model and direct effects

The structural model showed good fit: χ^2^(518) = 703.21, *p* < 0.001, CFI = 0.956, TLI = 0.952, RMSEA = 0.031, 90% CI [0.025, 0.037], SRMR = 0.045. The model explained 22.8% of creative self-efficacy, 33.4% of engagement, and 53.8% of final creative performance.

Before the mediators were included, motivation predicted final performance after baseline and background controls (β = 0.253, SE = 0.040, 95% CI [0.174, 0.332], *p* < 0.001), supporting H1 at the total-effect level. In the full model, motivation predicted creative self-efficacy (β = 0.448, *p* < 0.001) and engagement (β = 0.186, *p* < 0.001). Creative self-efficacy predicted engagement (β = 0.458, *p* < 0.001) and final performance (β = 0.163, *p* < 0.001). Engagement predicted final performance (β = 0.254, *p* < 0.001). The remaining direct effect of motivation was β = 0.080, *p* = 0.054, and was not statistically significant (see Table 7).

**Table 7:** Standardized direct effects.

Path	β	SE	95% CI	*p*	Decision
MLM → MCP total	0.253	0.040	[0.174, 0.332]	<0.001	H1 supported
MLM → CSE	0.448	0.047	[0.356, 0.540]	<0.001	H2 supported
CSE → MCP	0.163	0.046	[0.074, 0.252]	<0.001	H3 supported
MLM → LE	0.186	0.049	[0.090, 0.282]	<0.001	H4 supported
CSE → LE	0.458	0.049	[0.362, 0.554]	<0.001	H5 supported
LE → MCP	0.254	0.044	[0.168, 0.340]	<0.001	H6 supported
MLM → MCP direct	0.080	0.042	[−0.002, 0.162]	0.054	Not significant

MLM = music learning motivation; CSE = creative self-efficacy; LE = learning engagement; MCP = musical creative performance.

Baseline performance was the strongest covariate of final performance (β = 0.587, *p* < 0.001). Age (β = 0.028, *p* = 0.412), gender (β = 0.041, *p* = 0.264), music-major status (β = 0.052, *p* = 0.204), years of formal music training (β = 0.036, *p* = 0.279), weekly practice time (β = 0.049, *p* = 0.151), and previous composition experience (β = 0.057, *p* = 0.141) did not show statistically significant unique associations after baseline performance and the focal psychological variables were controlled. All prespecified covariates were retained regardless of statistical significance. Complete estimates are reported in Supplementary Table S1.

### 4.7 Indirect effects

The indirect effect through creative self-efficacy was β = 0.073, boot SE = 0.024, 95% CI [0.030, 0.123], supporting H7. The indirect effect through engagement was β = 0.047, boot SE = 0.014, 95% CI [0.022, 0.077], supporting H8. The sequential indirect effect through creative self-efficacy and engagement was β = 0.052, boot SE = 0.012, 95% CI [0.031, 0.078], supporting H9. The total indirect effect was β = 0.172, 95% CI [0.126, 0.223] (see Table 8).

**Table 8:** Bootstrap indirect effects.

Indirect pathway	Effect	Boot SE	Lower 95% CI	Upper 95% CI	Result
MLM → CSE → MCP	0.073	0.024	0.030	0.123	Significant
MLM → LE → MCP	0.047	0.014	0.022	0.077	Significant
MLM → CSE → LE → MCP	0.052	0.012	0.031	0.078	Significant
Total indirect effect	0.172	0.025	0.126	0.223	Significant

Contrasts among the three specific indirect effects were not significant, indicating complementary rather than competing processes. Creative self-efficacy was especially associated with originality and overall creative value, whereas engagement was most strongly associated with thematic development, coherence, and technical completion.

### 4.8 Robustness analyses and hypothesis summary

Full-information maximum likelihood estimation using all available longitudinal observations produced a sequential indirect association of β = 0.050, 95% CI [0.029, 0.075], closely matching the complete-case estimate. Excluding 28 highly experienced composers produced β = 0.049, 95% CI [0.027, 0.077]. The principal conclusions were also robust to covariate specification: when the model was re-estimated using baseline creative performance as the only covariate, the sequential indirect association remained significant (β = 0.053, 95% CI [0.032, 0.080]), while the residual direct association between motivation and final performance remained non-significant (β = 0.085, *p* = 0.052). Full comparisons are reported in Supplementary Table S2.

The conclusions were similarly robust to the composition of the musical creative performance score. When overall creative value was excluded and the outcome was recalculated using only originality, musical coherence, thematic development, expressiveness, and technical completion, creative self-efficacy (β = 0.159) and learning engagement (β = 0.249) remained positively associated with final performance. The sequential indirect association remained statistically significant (β = 0.051, 95% CI [0.030, 0.077]), and the residual direct association remained non-significant (β = 0.078, *p* = 0.062). Full comparisons are reported in Supplementary Table S3.

Students were nested within 12 classes in one institution (mean class size approximately 30.7). Class-level ICCs were small, ranging from 0.018 to 0.034 across the focal variables, with corresponding design effects ranging from 1.53 to 2.01. Re-estimation using class-clustered robust standard errors did not alter the substantive conclusions: the principal indirect pathways remained statistically significant, whereas the residual direct association between motivation and final performance remained non-significant. Clustering diagnostics and cluster-robust estimates are reported in Supplementary Tables S5 and S6 (see Table 9).

**Table 9:** Summary of hypothesis tests.

Hypothesis	Relationship	Result
H1	Motivation → creative performance	Supported at total-effect level
H2	Motivation → creative self-efficacy	Supported
H3	Creative self-efficacy → performance	Supported
H4	Motivation → engagement	Supported
H5	Creative self-efficacy → engagement	Supported
H6	Engagement → performance	Supported
H7	CSE mediates MLM–MCP	Supported
H8	Engagement mediates MLM–MCP	Supported
H9	Sequential mediation	Supported

## 5 Discussion

### 5.1 Overview

The findings were consistent with a model in which music learning motivation was prospectively associated with creative performance through subsequent creative self-efficacy and learning engagement. The total motivation-performance association was significant before the mediators were considered but was reduced to non-significance in the full model. Motivation predicted subsequent efficacy and engagement, creative self-efficacy predicted both later engagement and expert-rated performance, and engagement predicted the final products. The temporally ordered sequential indirect pathway was statistically supported.

### 5.2 Music learning motivation and creative performance

The positive total association is consistent with self-determination theory. Students who find music learning interesting and personally important may be more willing to explore possibilities, tolerate ambiguity, and persist through revision. These qualities are central to composition because a high-quality product rarely emerges from the first solution.

The reduction of the direct path after the mediators were included provides a more precise interpretation than the claim that motivated students are simply more creative. Motivation appears to be a relatively distal resource. A learner can value music without believing that successful composition is possible, and can feel motivated without investing sufficiently in a specific task. The present findings are therefore consistent with an indirect prospective association through capability beliefs and active involvement.

### 5.3 Creative self-efficacy

Music learning motivation strongly predicted subsequent creative self-efficacy. Motivated learners may accumulate mastery experiences, seek feedback, and interpret unsuccessful ideas as information rather than as evidence of fixed inability. These possible processes are consistent with social cognitive theory and with research identifying self-efficacy as an important predictor of motivation and engagement among music learners (Chen, 2024).

Creative self-efficacy also predicted expert-rated performance beyond baseline ability and engagement. The dimension-specific results suggested that it was especially relevant to originality and holistic creative value. Confidence may facilitate artistic risk-taking, the generation of alternatives, and persistence when an unfamiliar idea has not yet proved successful. Importantly, efficacy was measured by self-report while performance was independently rated, reducing the likelihood that the relationship reflected only common response tendencies.

### 5.4 Learning engagement

Learning engagement was positively associated with creative performance and was especially related to thematic development, coherence, and technical completion. These aspects require sustained work rather than confidence alone. Behavioral engagement creates time for practice and revision, emotional engagement supports personal involvement, and cognitive engagement supports monitoring and integration of musical decisions.

Engagement may therefore represent a behavioral pathway linking motivational and efficacy-related beliefs with subsequent creative performance. Motivation and efficacy reflect psychological readiness, while engagement captures the behavioral, emotional, and cognitive investment that accompanies the development of a musical product. This interpretation is consistent with the multidimensional engagement framework (Fredricks et al., 2004) and with evidence that musical creativity emerges dynamically through interaction and iterative development (Kupers and van Dijk, 2020).

### 5.5 Separate and sequential mediation

Creative self-efficacy showed a statistically significant indirect association between motivation and performance, indicating that higher motivation was associated with stronger subsequent task-specific beliefs about the capacity to produce original musical work. Engagement also showed a statistically significant indirect association, indicating that autonomous reasons for learning were associated with later active involvement. The mediators therefore represented distinct but related indirect pathways within the proposed temporal sequence.

The central contribution is the temporally ordered sequential pathway. Motivation represents reasons and direction; creative self-efficacy represents perceived capability; engagement represents active investment; and creative performance represents the demonstrated outcome. The sequence offers a theoretically coherent account of why motivation does not necessarily correspond directly to achievement. A student who values music but doubts creative capability may avoid open-ended work, while a confident student who does not persist or revise may still produce an underdeveloped product. These interpretations describe a prospective pattern of associations rather than a demonstrated causal process.

The four-wave order provides stronger temporal plausibility than a single-wave questionnaire but does not establish causality. Unmeasured factors, including teacher support, openness, achievement goals, and classroom climate, could influence the variables. The findings should therefore be interpreted as data consistent with the proposed sequence rather than proof of a causal chain.

### 5.6 Theoretical contributions

First, the model integrates self-determination theory, social cognitive theory, and engagement theory by locating each construct at a different point in a proposed creative-learning sequence. Second, it distinguishes motivation from subsequent psychological and behavioral processes through which motivation was prospectively associated with creative performance. Third, it separates perceived creative capability from independently evaluated creativity. Fourth, it controls baseline musical creative performance, thereby testing whether the psychological variables explain final performance beyond initial ability. Fifth, the dimension-specific analyses suggest that efficacy and engagement may be associated with different aspects of performance: efficacy was especially related to originality and holistic creative value, while engagement was especially related to elaboration and completion.

### 5.7 Educational implications

In university music-learning contexts similar to the one examined in the present study, educators may consider combining autonomy support, efficacy development, and opportunities for deep engagement. Autonomy-supportive practices include meaningful choices in style or instrumentation, explanations of task purpose, connection with learner interests, and acceptance of multiple solutions. Structure remains necessary, but it should be experienced as support rather than as unnecessary control. Recent qualitative research in Chinese university music classes similarly suggests that teacher scaffolding may support students’ autonomy, motivation, creativity, and a supportive learning environment, while also requiring sensitivity to learners’ differing needs and to the timing of instructional support (Li and Wang, 2026). In relation to the present findings, this evidence reinforces the potential value of process-focused feedback, guidance, and structured opportunities for revision while highlighting the importance of adapting such support to specific music-learning contexts.

Creative self-efficacy can be developed through attainable early challenges, progressive difficulty, specific process-focused feedback, peer models showing improvement, opportunities to revise, and explicit recognition of effective problem-solving. Feedback such as “the rhythmic transformation creates a clear contrast” supplies more useful efficacy information than global praise such as “you are creative.”

Engagement should be designed rather than assumed. Courses can use regular short creation tasks, staged deadlines, revision requirements, reflective journals, comparison of alternative versions, peer critique, and explanation of creative decisions. Assessment should consider both product and process, including expert evaluation, revision evidence, self-reflection, and documentation of decision-making.

Teachers should also avoid presenting creativity as a fixed talent. A developable view can encourage learners to interpret difficulty as a normal part of composition. This does not imply that every product is equally creative; it means that originality and musical effectiveness can improve through experimentation, feedback, strategy, and sustained work.

### 5.8 Limitations and future research

#### 5.8.1 Non-experimental design

Temporal separation and baseline adjustment strengthen plausibility but do not establish causality. Future studies should manipulate autonomy-supportive instruction or use quasi-experimental classroom designs.

#### 5.8.2 Incomplete longitudinal control

Each psychological construct was measured at one focal wave. Future research should measure all constructs repeatedly and use latent-change or random-intercept cross-lagged models.

#### 5.8.3 Self-reported mediators

Motivation, efficacy, and engagement remained self-reported. Behavioral indicators such as practice duration, revision frequency, persistence after feedback, and platform activity should be integrated.

#### 5.8.4 Task specificity

A notation-based composition task represents only one form of musical creativity. Future studies should include improvisation, arrangement, digital production, collaborative creation, and performance interpretation.

#### 5.8.5 Expert judgment

Multiple blinded experts and good ICCs reduce but do not eliminate subjectivity. Human judgment can be complemented by process data, audience response, and carefully interpreted computational indicators.

#### 5.8.6 Sample and context

Participants were undergraduate students enrolled in music-related courses at one university in Jinan, China. This institutional sample should not be interpreted as representative of Chinese university music students as a whole, and the findings may not generalize to younger learners, professional musicians, informal music learners, or students in other regional and institutional contexts. Replication across universities, regions, countries, and forms of music learning is needed. Clustered-data sensitivity analyses were based on only 12 classes; although the class-clustered robust estimates produced the same substantive conclusions, the relatively small number of clusters warrants cautious interpretation of the cluster-adjusted standard errors.

#### 5.8.7 Reciprocal relationships

Creative success may strengthen later self-efficacy, engagement, and motivation. Longer longitudinal research should examine reciprocal developmental dynamics.

#### 5.8.8 Motivation quality

The principal model focused on autonomous motivation. Future studies should compare autonomous and controlled pathways and examine whether external pressure has different implications for risk-taking and creativity.

## 6 Conclusion

This study examined a four-wave, multi-source framework linking music learning motivation with subsequent musical creative performance through creative self-efficacy and learning engagement. Autonomous motivation showed a positive total association with expert-rated performance, but the remaining direct association was not statistically significant after creative self-efficacy and engagement were considered.

Statistically significant indirect associations were observed through creative self-efficacy and learning engagement, including the proposed temporally ordered sequential pathway from motivation to efficacy, from efficacy to engagement, and from engagement to performance. The findings were consistent with the interpretation that learners who value and enjoy music tend to report stronger subsequent confidence in their creative capability, that this confidence is associated with deeper behavioral, emotional, and cognitive involvement, and that sustained involvement is associated with more original, coherent, expressive, and technically developed musical products. These results should be interpreted as prospective associations rather than evidence of a causal mediation mechanism.

For music education, the findings highlight the potential relevance of autonomy-supportive learning environments, attainable creative success, process-focused feedback, structured opportunities for revision, and active creation. However, the present findings are specific to an institutional sample drawn from one Chinese university. Because the design was prospective rather than experimental, the observed relationships should be interpreted as temporally ordered associations rather than causal effects. Replication across universities, regions, educational levels, and forms of music learning is needed before broader generalization is warranted.

## Data Availability

The raw data supporting the conclusions of this article will be made available by the authors, without undue reservation.
