# Supplementary material for: Music learning motivation and students’ musical creative performance: the sequential mediating roles of creative self-efficacy and learning engagement
Source: Front Psychol. 2026 Sep 4;17:1949126. doi: 10.3389/fpsyg.2026.1949126 (PMC13585972; doi:10.3389/fpsyg.2026.1949126)
Supplement: Supplementary file 1 [file Supplementary_file_1.pdf]

Appendix A. Four-Wave Data Collection Schedule

Table A1. Data collection schedule

Wave	Approximate timing	Primary measures or task	Expected duration
T1	Week 1	Consent, background, motivation, baseline creation task	75 minutes
T2	Week 4	Creative self-efficacy	5–8 minutes
T3	Week 7	Behavioral, emotional, and cognitive engagement	8–10 minutes
T4	Week 10	Parallel final creation task	60 minutes
Rating	After T4	Blind expert evaluation of mixed T1/T4 products	Independent

Appendix B. Expert Rating Anchors

Each dimension is rated from 1 to 7. Raters should judge creative quality rather than personal liking or stylistic preference. A score of 1 represents a very limited demonstration of the dimension, 4 represents an adequate or moderate demonstration, and 7 represents an exceptional demonstration.

Table B1. Condensed scoring anchors

Dimension	1	4	7
Originality	Repetitive or derivative	Several individual choices	Exceptionally original and meaningful
Coherence	Fragmented and difficult to follow	Generally coherent but uneven	Exceptional unity and continuity
Thematic development	No meaningful development	Adequate recognizable development	Sophisticated integration and transformation
Expressiveness	No clear expressive direction	Moderately clear character	Exceptional expressive depth
Technical completion	Incomplete or unusable	Generally complete with minor weaknesses	Exceptional technical control
Overall creative value	Very limited creative merit	Moderate overall creative quality	Exceptional creative achievement

Supplementary Material

The following tables provide expanded statistical reporting requested during peer review.

Table S1. Covariate effects in the fully adjusted structural model

Covariate → T4 MCP	β	SE	95% CI	p
Baseline MCP	.587	.041	[.507, .667]	<.001
Age	.028	.034	[-.039, .095]	.412
Gender	.041	.037	[-.031, .113]	.264
Music-major status	.052	.041	[-.028, .132]	.204
Years of formal music training	.036	.033	[-.029, .101]	.279
Weekly practice time	.049	.034	[-.018, .116]	.151
Previous composition experience	.057	.039	[-.019, .133]	.141

Note. MCP = musical creative performance. All covariates were retained in the fully adjusted model irrespective of statistical significance.

Table S2. Sensitivity of principal effects to covariate specification

Effect	Fully adjusted β	Fully adjusted 95% CI	Baseline-only β	Baseline-only 95% CI
MLM → CSE	.448	[.356, .540]	.451	[.360, .542]
MLM → LE	.186	[.090, .282]	.190	[.095, .285]
CSE → LE	.458	[.362, .554]	.456	[.361, .551]
CSE → MCP	.163	[.074, .252]	.168	[.079, .257]
LE → MCP	.254	[.168, .340]	.258	[.172, .344]
MLM → MCP direct	.080	[-.002, .162]	.085	[-.001, .171]
MLM → CSE → MCP	.073	[.030, .123]	.076	[.032, .127]
MLM → LE → MCP	.047	[.022, .077]	.049	[.023, .080]
MLM → CSE → LE → MCP	.052	[.031, .078]	.053	[.032, .080]
Total indirect effect	.172	[.126, .223]	.178	[.131, .230]

Note. The baseline-only model retained baseline musical creative performance as the sole covariate.

Table S3. Sensitivity analysis excluding overall creative value from the performance composite

Effect	Six-dimension β	Six-dimension 95% CI	Five-dimension β	Five-dimension 95% CI
CSE → MCP	.163	[.074, .252]	.159	[.069, .249]
LE → MCP	.254	[.168, .340]	.249	[.162, .336]
MLM → MCP direct	.080	[-.002, .162]	.078	[-.004, .160]
MLM → CSE → MCP	.073	[.030, .123]	.071	[.028, .121]
MLM → LE → MCP	.047	[.022, .077]	.046	[.021, .076]
MLM → CSE → LE → MCP	.052	[.031, .078]	.051	[.030, .077]
Total indirect effect	.172	[.126, .223]	.168	[.122, .219]

Note. The five-dimension score included originality, musical coherence, thematic development, expressiveness, and technical completion, but excluded overall creative value.

Table S4. Standardized factor loadings for higher-order self-report constructs and musical creative performance

Higher-order/common construct	Indicator	Standardized loading
Autonomous motivation	Intrinsic motivation	.92
Autonomous motivation	Identified regulation	.89
Learning engagement	Behavioral engagement	.87
Learning engagement	Emotional engagement	.90
Learning engagement	Cognitive engagement	.93
Musical creative performance	Originality	.82
Musical creative performance	Musical coherence	.86
Musical creative performance	Thematic development	.84
Musical creative performance	Expressiveness	.74
Musical creative performance	Technical completion	.79
Musical creative performance	Overall creative value	.90

Table S5. Class-level clustering diagnostics

Variable	ICC	Mean cluster size	Design effect
Music learning motivation	.021	30.7	1.62
Creative self-efficacy	.026	30.7	1.77
Learning engagement	.031	30.7	1.92
Baseline MCP	.018	30.7	1.53
Final MCP	.034	30.7	2.01

Note. Participants were nested in 12 classes within one institution; the analytical sample was N = 368.

Table S6. Class-clustered robust estimates for principal structural paths

Path	β	Cluster-robust SE	p
MLM → CSE	.448	.052	<.001
MLM → LE	.186	.054	.001
CSE → LE	.458	.053	<.001
CSE → MCP	.163	.051	.001
LE → MCP	.254	.049	<.001
MLM → MCP direct	.080	.046	.082

Note. The class-clustered sequential indirect association was β = .052, 95% CI [.028, .080].
